# Association Between Healthy Eating Index-2015 and Kidney Stones in American Adults: A Cross-Sectional Analysis of NHANES 2007–2018

**DOI:** 10.3389/fnut.2022.820190

**Published:** 2022-05-24

**Authors:** Shan Yin, Jiahao Wang, Yunjin Bai, Zhenzhen Yang, Jianwei Cui, Yunfei Xiao, Jia Wang

**Affiliations:** ^1^Department of Urology, Institute of Urology, West China Hospital, Sichuan University, Chengdu, China; ^2^Department of Clinical Laboratory, Nanchong Central Hospital, Nanchong, China

**Keywords:** kidney stones, HEI-2015, NHANES, association, cross-sectional analysis

## Abstract

**Purpose:**

To explore the association between Healthy Eating Index (HEI)-2015 and kidney stones in an American adult population.

**Materials and Methods:**

National Health and Nutrition Examination Survey (NHANES) datasets from 2007 to 2018 were used. Participants aged ≥ 20 years who reported kidney stone history and dietary recall were included. Weighted proportions, multivariable analysis and spline smoothing were used to evaluate the associations between HEI-2015 and nephrolithiasis by adjusting gender, age, race, poverty income ratio, body mass index, education level, marital status, smoking, alcohol intake, energy level, vigorous activity, moderate activity, and some comorbidities.

**Results:**

Totally 30 368 American adults were included, with weighted mean age [standard deviation (SD)] of 47.69 (16.85) years. The overall mean HEI-2015 score (SD) was 50.82 (13.80). In the fully-adjusted multivariable model, HEI-2015 was negatively correlated with urolithiasis [odds ratio (OR) = 0.991; 95% confidence interval (CI) 0.988 to 0.994]. Compared with the first quartile of HEI-2015, the population in the fourth quartile of HEI-2015 had a lower prevalence of kidney stones (OR = 0.716; 95% CI 0.635 to 0.807). The association was modified by education and vigorous activity.

**Conclusions:**

HEI-2015 is inversely associated with the prevalence of kidney stones, which means better diet quality is associated with a lower risk of nephrolithiasis.

## Introduction

Kidney calculus is a common disease worldwide and easily recurs after treatment, which puts heavy burden on the medical and health system. The formation of kidney stones is a multi-factorial process that may involve genetic, metabolic, anatomical and functional abnormalities, and is vitally affected by nutrition (1). A variety of foods or diets are related to kidney stones, such as fruit juices and fruit juice beverages, soft drinks, tea, coffee (1), and the Mediterranean diet (2). Many nutrient components are also associated with kidney calculi, including protein, fat, and carbohydrates (1). However, there is little research on elucidating the association between diet quality and kidney stones.

Dietary behavior is an extremely important lifestyle for individuals, as diet is related not only to the maintenance of normal physiological functions in the body, but also to many diseases (3, 4). The Healthy Eating Index (HEI) is a diet quality measure used to assess how well a set of foods aligns with key recommendations of the Dietary Guidelines for Americans (5). HEI can quantitatively assess the diet quality of various human groups and examine the prospective and cross-sectional links of diet quality with health outcomes, such as offspring overweight (6), risk for all-cause mortality of cardiovascular diseases and cancers (7), and depression (8). HEI-2015 as the most current version meets the main recommendations of the 2015–2020 Dietary Guidelines for Americans (5).

But there is limited evidence about the relationship between overall diet quality and kidney stones. Therefore, we investigated the association between HEI-2015 and nephrolithiasis by analyzing National Health and Nutrition Examination Survey (NHANES) cross-sectional data. We hypothesize that better diet quality, namely higher HEI-2015, is associated with lower prevalence of kidney stones.

## Materials and Methods

### Data Source and Population

**Figure 1:**
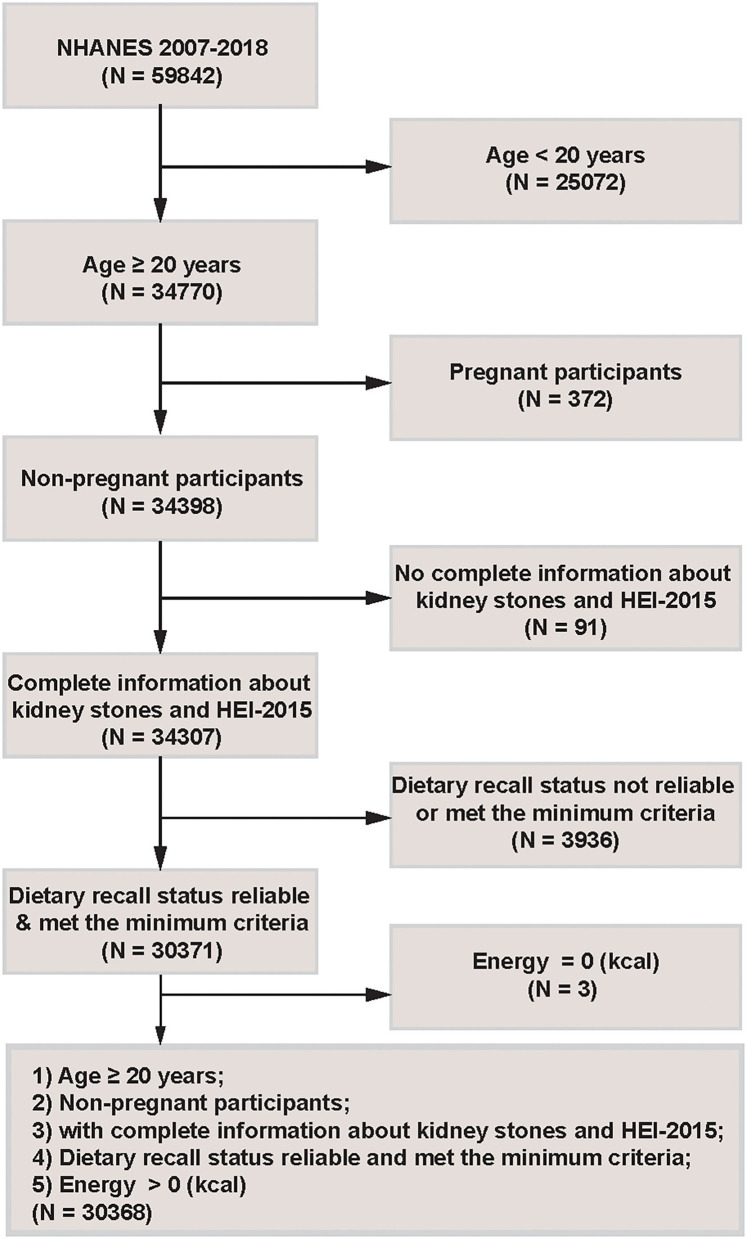
Flowchart of the study population.

NHANES is a program of studies designed to evaluate participants' health and nutritional status in America. It combines interviews and physical examinations. We used 6 continuous cycles of NHANES data from year 2007 to 2018. This cross-sectional study included participants aged 20 years or older (*n* = 34 770). Then pregnant participants (*n* = 372) and people with incomplete information about kidney stones and HEI-2015 (*n* = 91) were excluded. Moreover, participants under unreliable dietary recall status or below the levels of minimum criteria (*n* = 3,936), or with energy intake equal to 0 kcal (*n* = 3) were excluded. Finally, 30 368 eligible people were included for further analyses. The detailed inclusion and exclusion criteria were shown in Figure 1.

All NHANES study protocols were approved by the National Center for Health Statistics (NCHS) research ethics review board and by all participants.

### Outcome and Exposure Factors

The main outcome was if one person ever had nephrolithiasis. In NHANES questionnaire sections, participants who replied “yes” to “Have you/Has sample person (SP) ever had kidney stones?” were considered to have a history of kidney calculi. Namely, kidney stone was identified through the participants' self-reports.

The major exposure factor was HEI-2015. HEI-2015 was designed and scored from 0 to 100, which was derived from the sum of 13 components. First, nine adequacy components include total fruits, whole fruits, total vegetables, greens and beans, total protein foods, seafood and plant proteins (each 0–5 points); whole grains, dairy, and fatty acids (each 0–10 points). Second, four moderation components are sodium, refined grains, added sugars, and saturated fats (each 0–10 points) (5). A higher HEI-2015 score indicates better diet quality. The 13 components of HEI-2015 were calculated using the total nutrient intakes on the first day (DR1TOT), the second day (DR2TOT) and United States Department of Agriculture (USDA) MyPyramid Equivalents Database/Food Patterns Equivalents Database (MPED/FPED) files. All the analysis of the diet quality was carried out by ourselves. Supplementary Table S1 showed the detailed HEI-2015 scoring criteria.

### Covariates

To make the association between kidney stones and HEI-2015 robust, we adjusted the following covariates: age, gender, race, marital status, education, poverty income ratio, body mass index (BMI), smoking, alcohol, energy; vigorous activity, moderate activity, and some self-reported medical conditions (all classified as yes/no). Age was categorized as 20–35, 35–50, 50–65, and >65 years. Races included Mexican American, other Hispanic, non-Hispanic white, non-Hispanic black, other races. Income ratio was set at ≤ 1.3, >1.3 and ≤ 3.5, >3.5. BMI was divided as <25, 25–30, and ≥30 kg/m^2^. Smoking was classified as <100 (non-smoker) or ≥100 cigarettes (smoker) in life. Alcohol was defined as <12 (non-drinker) or ≥12 alcohol drinks (drinker) per year. Marital status was divided into married, widowed, divorced, separated, never married, and living with partner. Education level included < 9th grade, 9–11th grade, high school graduate, some college, and college graduate or above. The medical conditions included gout, diabetes, high blood pressure, congestive heart failure, and cancer. Dummy variables were used to indicate missing covariate values for variables with missing values > 2%.

### Statistical Analysis

We used sampling weights recommended by Centers for Disease Control and Prevention (CDC). In the baseline characteristics table, continuous variables were expressed as mean ± standard deviation (SD), and categorical variables as proportions. For the two types of variables, differences among HEI-2015 quartiles were examined by survey-weighted linear regression and survey-weighted Chi-square test respectively. And the *P*-value for trend was obtained by fitting a logistic regression model.

To explore the association between HEI-2015 and kidney stones, we used three logistic regression models with or without adjustment of covariates. Model 1 was unadjusted. Model 2 was adjusted for age, gender and race. Model 3 was adjusted for gender, age, race, poverty income ratio, BMI, education, marital status, smoking, alcohol, energy, vigorous activity, moderate activity, gout, diabetes, high blood pressure, congestive heart failure, and cancer. The same method was used to explore the relationships between the 13 HEI-2015 components and nephrolithiasis. Then we did three sensitivity analyses: 1. We removed the HEI-2015 extreme values that were less than mean-3SD and greater than mean+3SD, and then followed the above-mentioned multivariate logistic regression model to analyze the relationship between HEI-2015 and kidney stones; 2. We removed individuals with very low or high caloric intake (<800 or >4,200 kcal/d in men and <600 or >3,500 kcal/d in women), and then conducted a sensitivity analysis; 3. Furthermore, we calculated the HEI-2015 scores based on the day 2 dietary recall data, and then performed a sensitivity analysis.

To better explore the association between HEI-2015 and kidney stones, multivariable logistic regression was conducted to explore HEI-2015 as continuous and categorical variables (divided into quarters). The trends were estimated by treating HEI-2015 quartiles as a continuous variable. Then to test whether there was a non-linear association between HEI-2015 and kidney stones, we performed spline smoothing with a generalized additive model (GAM). Finally, we further used stratified logistic regression models for sensitive analyses according to all potential confounding factors at the baseline table.

We combined the sample weights of 6 continuous cycles according to the recommended method on the NHANES website (https://www.cdc.gov/nchs/nhanes/index.htm). According to the suggestion, a variable of interest that was collected on the smallest number of respondents was the “least common denominator.” The sample weight that applies to that variable was the appropriate one to use for our analysis. All analyses were performed using R (http://www.R-project.org; The R Foundation) and EmpowerStats (http://www.empowerstats.com, X&Y Solutions, Inc.). We considered a 2-tailed *P* < 0.05 as statistically significant.

## Results

### Population Characteristics

Figure 1 described the study design, inclusion and exclusion criteria. A total of 30 368 American adults were included, with weighted mean age (SD) of 47.69 (16.85) years, 48.65% males and 51.35% females. In the fourth quartile of the HEI-2015 population, participants with college level or above accounted for the maximum ratio compared with the other three quartiles. Table 1 presented the baseline population characteristics according to the quartiles of HEI-2015.

**Table 1:** Characteristics of participants by categories of Healthy Eating Index 2015: NHANES 2007–2018.

	**All**	**Q1(8.81–40.89)**	**Q2(40.89–50.33)**	**Q3(50.34–60.35)**	**Q4(60.35–97.88)**	***P*** **-value**	***P*** for trend^*^
Non-weighted N	30,368	7,592	7,592	7,592	7,592		
HEI_2015	50.82 ± 13.80	33.91 ± 5.20	45.68 ± 2.73	55.07 ± 2.88	69.22 ± 7.06	<0.001	<0.001
Energy (kcal)	2168.86 ± 990.76	2262.99 ± 1102.17	2231.38 ± 1011.26	2154.57 ± 969.63	2022.48 ± 840.47	<0.001	<0.001
Age (years, mean ± SD)	47.69 ± 16.85	43.85 ± 16.32	46.69 ± 16.44	48.89 ± 16.89	51.45 ± 16.81	<0.001	<0.001
20–34 (%)	26.88	34.84	27.82	23.94	20.66		<0.001
35–49 (%)	27.44	29.13	29.43	27.93	23.16		<0.001
50–64 (%)	27.16	23.56	26.71	27.61	30.86		<0.001
≥65 (%)	18.53	12.47	16.04	20.53	25.32		<0.001
Gender (%)						<0.001	
Male	48.65	52.77	50.86	47.87	42.92		<0.001
Female	51.35	47.23	49.14	52.13	57.08		<0.001
Race (%)						<0.001	
Mexican American	8.33	8.47	8.82	8.82	7.20		0.025
Other Hispanic	5.69	5.33	5.54	5.88	6.03		0.070
Non-Hispanic white	67.21	67.19	66.73	66.86	68.08		0.505
Non-Hispanic black	11.18	13.05	12.29	10.70	8.61		<0.001
Other races	7.58	5.97	6.61	7.73	10.07		<0.001
Education (%)						<0.001	
Less than 9th grade	5.13	4.92	5.20	5.75	4.66		0.851
9–11th grade	10.32	13.74	11.05	9.65	6.73		<0.001
High school graduate	23.14	28.75	25.26	22.01	16.35		<0.001
Some college	31.46	32.76	33.18	31.86	27.96		<0.001
College graduate or above	29.95	19.83	25.32	30.73	44.30		<0.001
Marital Status (%)						<0.001	
Married	55.17	48.89	54.49	56.22	61.26		<0.001
Widowed	5.61	4.09	4.77	6.84	6.82		<0.001
Divorced	10.30	11.00	11.04	10.45	8.67		0.001
Separated	2.40	2.60	2.54	2.57	1.88		0.017
Never married	18.37	23.12	18.78	16.63	14.82		<0.001
Living with partner	8.15	10.30	8.39	7.29	6.55		<0.001
Poverty Income Ratio (mean ± SD)						<0.001	<0.001
≤ 1.3 (%)	20.01	25.67	21.26	18.64	14.26		<0.001
>1.3 and ≤ 3.5 (%)	33.09	35.85	33.64	33.62	29.14		<0.001
>3.5 (%)	39.68	31.55	38.48	40.48	48.46		<0.001
Missing (%)	7.23	6.93	6.61	7.27	8.14		0.027
BMI (kg/m^2^, mean ± SD)	29.13 ± 6.92	30.12 ± 7.46	29.57 ± 7.10	28.99 ± 6.68	27.79 ± 6.12	<0.001	<0.001
<25 (%)	27.84	23.93	26.06	27.50	34.04		<0.001
25–30 (%)	1.55	1.76	1.48	1.41	1.56		0.464
≥30 (%)	32.69	30.01	31.44	34.25	35.16		<0.001
Missing (%)	37.92	44.29	41.02	36.85	29.25		<0.001
Smoking (%)						<0.001	
Non-smoker	55.69	50.24	53.18	57.26	62.27		<0.001
Smoker	44.31	49.76	46.82	42.74	37.73		<0.001
Alcohol (%)						<0.001	
Non-drinker	17.76	17.46	16.42	17.79	19.42		0.009
Drinker	60.65	59.62	61.32	61.34	60.32		0.694
Missing	21.59	22.92	22.26	20.87	20.26		0.133
High Blood Pressure (%)						0.115	
No	67.66	68.69	67.33	66.93	67.68		0.316
Yes	32.34	31.31	32.67	33.07	32.32		0.316
Diabetes (%)						0.034	
No	87.91	88.80	87.81	87.63	87.37		0.043
Yes	9.89	9.33	9.78	10.30	10.17		0.112
Missing	2.20	1.88	2.41	2.06	2.46		0.177
Congestive Heart Failure (%)						0.063	
No	97.63	97.84	97.64	97.24	97.80		0.533
Yes	2.37	2.16	2.36	2.76	2.20		0.533
Cancer (%)						<0.001	
No	89.54	91.43	89.74	88.97	87.97		<0.001
Yes	10.46	8.57	10.26	11.03	12.03		<0.001
Gout (%)						0.187	
No	95.88	95.65	96.18	96.08	95.63		0.910
Yes	4.12	4.35	3.82	3.92	4.37		0.910
Vigorous activity (%)						<0.001	
No	73.99	78.45	77.07	73.80	66.46		<0.001
Yes	26.01	21.55	22.93	26.20	33.54		<0.001
Moderate activity (%)						<0.001	
No	54.19	62.28	57.60	53.43	43.15		<0.001
Yes	45.81	37.72	42.40	46.57	56.85		<0.001
Kidney Stones (%)						<0.001	
No	89.98	89.19	88.94	90.13	91.71		<0.001
Yes	10.02	10.81	11.06	9.87	8.29		<0.001

*Mean ± SD for continuous variables, P-value was by survey-weighted linear regression*.

*% for categorical variables, P-value was by survey-weighted Chi-square test*.

* *P for trend was by fitting a logistic regression model*.

People ever having kidney stones were elder (mean ± SD = 53.60 ± 15.45) than those not having kidney stones (47.03 ± 16.87). Participants with nephrolithiasis had a higher BMI (30.65 ± 6.96) than non-stone participants (28.96 ± 6.89) (Supplementary Table S2). Besides, a less proportion of kidney stone participants had vigorous and moderate activity than the non-calculus people.

### Outcome and Exposure Factors

According to the quartiles of HEI-2015, nephrolithiasis ever accounted for 10.81%, 11.06%, 9.87%, and 8.29% in Q1 (8.81–40.89), Q2 (40.89–50.33), Q3 (50.34–60.35), and Q4 (60.35–97.88), respectively. The overall prevalence of kidney stones was 10.02%. The overall mean (SD) HEI-2015 score was 50.82 (13.80). Supplementary Figure S1 presented the ratios of mean scores of HEI-2015 components to maximum scores, prevalence of kidney stones and HEI-2015 mean scores in each NHANES cycle. The weighted prevalence of nephrolithiasis was 9.13% in 2007–2008, 8.99% in 2009–2010, 8.70% in 2011–2012, 10.46% in 2013–2014, 11.93% in 2015–2016, and 10.76% in 2017–2018. The non-stone people had a higher mean HEI-2015 score than the participants with kidney stones (51.00 ± 13.88 vs. 49.24 ± 13.01) (Supplementary Table S2). Supplementary Table S3 showed the distributions of HEI-2015 components by categories of HEI-2015.

### Multivariate Regression Analysis

Multivariate regression analysis showed that HEI-2015 was negatively correlated with urolithiasis in the non-adjusted model (model 1) (OR =0.992; 95%CI 0.990 to 0.995), the minimally adjusted model (model 2) (OR = 0.988; 95%CI 0.985 to 0.991), and the fully adjusted model (model 3) (OR = 0.991; 95%CI 0.988 to 0.994). Based on the quartiles of HEI-2015, all three models showed a negative correlation between HEI-2015 and the prevalence of kidney stones. Namely, higher HEI-2015 was associated with lower prevalence of nephrolithiasis. Compared with the first quartile of the HEI-2015 population, the fourth quartile had a lower kidney stone prevalence in model 1 (OR = 0.771; 95%CI 0.690 to 0.862), model 2 (OR = 0.661; 95%CI 0.589 to 0.741) and model 3 (OR = 0.716; 95%CI 0.635 to 0.807). *P* for trend was < 0.001 in all three models (Table 2).

**Table 2:** Association of Healthy Eating Index 2015 with kidney stones.

**Exposure**	**Model 1^**a**^**	**Model 2^**b**^**	**Model 3^**c**^**
HEI-2015 (continuous)	0.992 (0.990, 0.995) <0.001	0.988 (0.985, 0.991) <0.001	0.991 (0.988, 0.994) <0.001
Quartile of HEI-2015			
Q1(8.81–40.89)	1.0	1.0	1.0
Q2(40.89–50.33)	1.010 (0.910, 1.122) 0.852	0.963 (0.865, 1.071) 0.485	0.985 (0.884, 1.099) 0.792
Q3(50.34–60.35)	0.944 (0.849, 1.050) 0.291	0.853 (0.765, 0.951) 0.004	0.896 (0.802, 1.002) 0.055
Q4(60.35–97.88)	0.771 (0.690, 0.862) <0.001	0.661 (0.589, 0.741) <0.001	0.716 (0.635, 0.807) <0.001
*P*-value for trend	<0.001	<0.001	<0.001

a *Non-adjusted model: adjusted for None*.

b *Minimally adjusted model: adjusted for gender, age, race*.

c *Fully adjusted model: adjusted for gender, age, race, poverty income ratio, BMI, education, marital status, smoking, alcohol, energy, vigorous activity, moderate activity, gout, diabetes, high blood pressure, congestive heart failure, cancer*.

Multivariate regression analysis of HEI-2015 components demonstrated that scores of total fruits, whole fruits, total vegetables, added sugars, saturated fats, whole grains and dairy were all significantly associated with kidney stones. The most associated components included added sugars (OR = 0.960; 95%CI 0.949 to 0.972), total fruits (OR = 0.968; 95%CI 0.949 to 0.987), total vegetables (OR = 0.969; 95%CI 0.946 to 0.992), and whole fruits (OR = 0.970; 95%CI 0.952 to 0.987) (details in Table 3).

**Table 3:** Association of HEI-2015 components with kidney stones.

**HEI-2015 Components**	**Model 1^**a**^**	**Model 2^**b**^**	**Model 3^**c**^**
Adequacy components			
Total fruits	0.975 (0.958, 0.993) 0.007	0.955 (0.937, 0.974) <0.001	**0.968 (0.949, 0.987) 0.001**
Whole fruits	0.986 (0.969, 1.002) 0.094	0.960 (0.943, 0.977) <0.001	**0.970 (0.952, 0.987)** ** <0.001**
Total vegetables	0.983 (0.961, 1.006) 0.139	0.962 (0.941, 0.985) 0.001	**0.969 (0.946, 0.992) 0.009**
Greens and beans	0.976 (0.959, 0.994) 0.008	0.979 (0.961, 0.997) 0.023	0.990 (0.972, 1.009) 0.304
Whole grains	1.000 (0.988, 1.011) 0.953	0.982 (0.971, 0.994) 0.004	0.984 (0.972, 0.997) 0.013
Dairy	0.998 (0.986, 1.009) 0.665	0.983 (0.972, 0.995) 0.005	0.986 (0.975, 0.998) 0.021
Total protein foods	0.983 (0.954, 1.012) 0.246	0.978 (0.949, 1.008) 0.144	0.972 (0.942, 1.002) 0.068
Seafood and plant proteins	0.984 (0.967, 1.000) 0.054	0.976 (0.959, 0.993) 0.005	0.987 (0.970, 1.005) 0.159
Fatty acids	0.984 (0.974, 0.995) 0.003	0.992 (0.981, 1.003) 0.134	0.994 (0.984, 1.005) 0.312
Moderation components			
Sodium	0.995 (0.984, 1.006) 0.365	0.994 (0.983, 1.005) 0.297	1.003 (0.992, 1.014) 0.607
Refined grains	1.001 (0.991, 1.011) 0.879	0.990 (0.980, 1.001) 0.067	0.993 (0.982, 1.004) 0.213
Added sugars	0.981 (0.970, 0.992) <0.001	0.964 (0.953, 0.975) <0.001	**0.960 (0.949, 0.972)** ** <0.001**
Saturated fats	0.966 (0.955, 0.976) <0.001	0.976 (0.965, 0.987) <0.001	0.984 (0.973, 0.996) 0.007

a *Non-adjusted model adjusted for: None*.

b *Adjust I model adjust for: gender, age, race*.

c *Adjust II model adjust for: gender, age, race, poverty income ratio, BMI, education, marital status, smoking, alcohol, energy, vigorous activity, moderate activity, gout, diabetes, high blood pressure, congestive heart failure, cancer. Bold values indicate four smallest effect sizes (OR)*.

### Spline Smoothing

**Figure 2:**
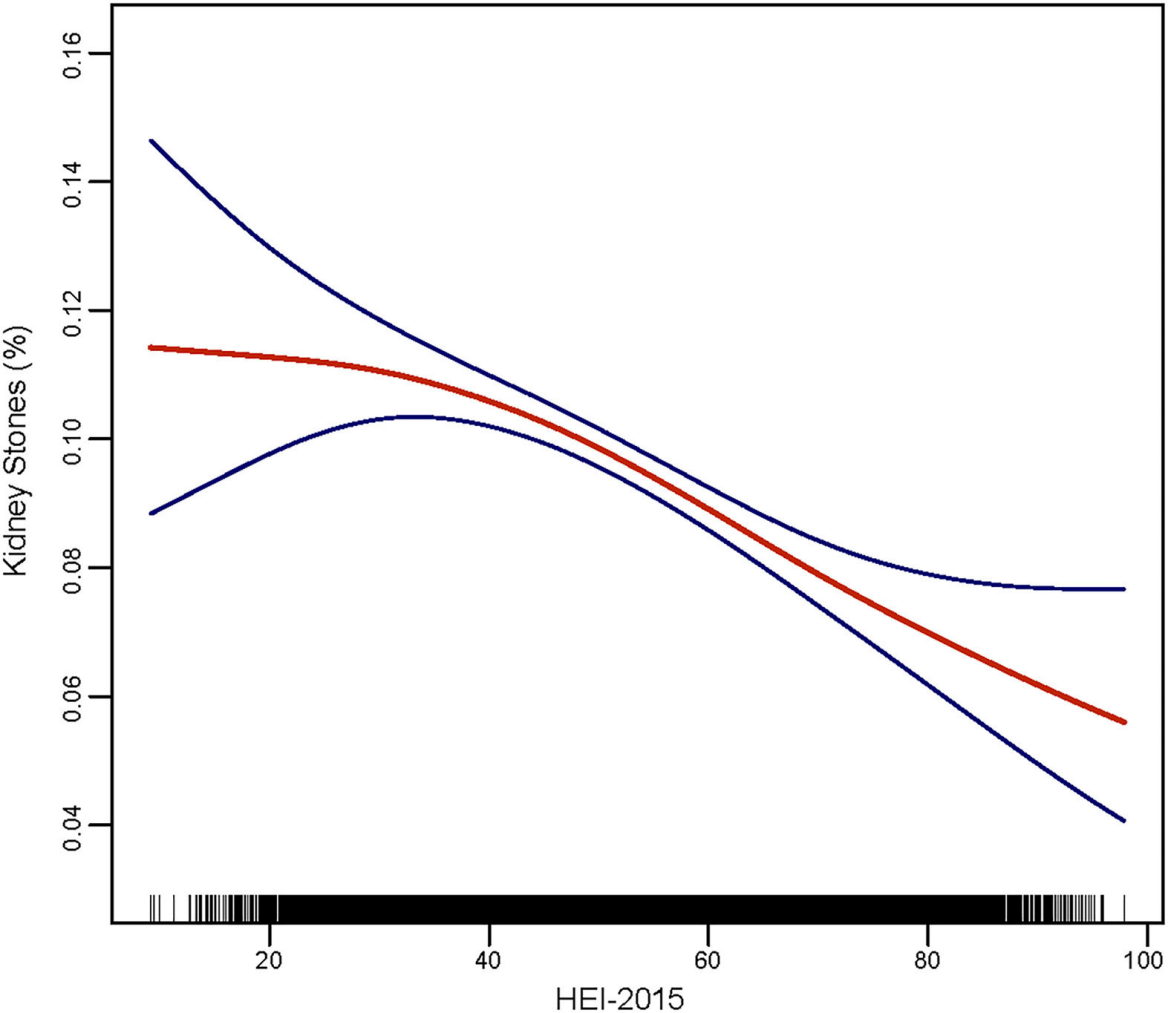
Smooth curve fitting of kidney stones and HEI-2015.

Smooth curve fitting was performed to explore the non-linear association between HEI-2015 and nephrolithiasis (Figure 2), which showed a fully-adjusted smooth curve. From this curve, we can regard a linear relationship between HEI-2015 and the prevalence of kidney stones. Higher HEI-2015 was associated with fewer kidney stones, which indicated a negative association. Compared to the first third of the line, the remaining parts of this line had a greater slope.

### Analyses of Subgroups and Interactions

Subgroup analyses (Table 4) found that education and vigorous activity were effect modifiers for the relationship between HEI-2015 and kidney stones after adjustment of other covariates. Results showed the effect sizes of the relationship in different education levels and vigorous activity status were significantly different. As for the education level, the odds ratios of people with < 9th grade, 9–11th grade, high school graduate, some college, and college graduate or above were 0.999, 0.996, 0.994, 0.988, and 0.985, respectively with *p* of 0.0414 for interaction. For the vigorous activity status, the odds ratios of participants with and without vigorous activity were 0.984 and 0.992, respectively, with *p* of 0.0392 for interaction.

**Table 4:** Stratifiedlogistic regression analysis to identify variables that modify the correlation between HEI-2015 and kidney stones^a^.

	**Model 1^***a***^**	***P*** **for interaction**	**Model 2^***b***^**	***P*** **for interaction**	**Model 3^***c***^**	***P*** **for interaction**
Gender		0.064		0.542		0.837
Male	0.996 (0.992, 0.999)		0.989 (0.985, 0.993)		0.990 (0.986, 0.994)	
Female	0.990 (0.986, 0.994)		0.987 (0.983, 0.991)		0.991 (0.986, 0.995)	
Age (years)		0.390		0.440		0.883
20–34	0.987 (0.979, 0.996)		0.987 (0.978, 0.995)		0.990 (0.981, 0.999)	
35–49	0.985 (0.979, 0.991)		0.986 (0.980, 0.992)		0.991 (0.984, 0.997)	
50–64	0.986 (0.981, 0.991)		0.986 (0.981, 0.991)		0.989 (0.984, 0.994)	
≥65	0.991 (0.986, 0.996)		0.991 (0.986, 0.996)		0.992 (0.987, 0.997)	
Race		0.497		0.530		0.921
Mexican American	0.999 (0.991, 1.007)		0.994 (0.986, 1.002)		0.994 (0.986, 1.002)	
Other Hispanic	0.994 (0.986, 1.002)		0.990 (0.982, 0.998)		0.991 (0.983, 1.000)	
Non-Hispanic white	0.991 (0.987, 0.995)		0.987 (0.983, 0.990)		0.990 (0.986, 0.994)	
Non-Hispanic black	0.994 (0.986, 1.002)		0.990 (0.982, 0.998)		0.990 (0.982, 0.998)	
Other races	0.991 (0.982, 1.000)		0.986 (0.976, 0.995)		0.993 (0.982, 1.003)	
Education		0.024		0.005		0.041
Less than 9th grade	0.999 (0.991, 1.008)		0.999 (0.990, 1.008)		0.999 (0.989, 1.009)	
9–11th grade	0.996 (0.989, 1.004)		0.994 (0.986, 1.002)		0.996 (0.988, 1.004)	
High school graduate	0.997 (0.991, 1.004)		0.993 (0.986, 0.999)		0.994 (0.988, 1.001)	
Some college	0.992 (0.987, 0.997)		0.986 (0.981, 0.991)		0.988 (0.982, 0.993)	
College graduate or above	0.985 (0.980, 0.991)		0.981 (0.975, 0.987)		0.985 (0.979, 0.991)	
Marital Status		0.120		0.092		0.090
Married	0.988 (0.984, 0.991)		0.986 (0.982, 0.990)		0.988 (0.984, 0.992)	
Widowed	0.999 (0.990, 1.008)		1.000 (0.991, 1.009)		1.002 (0.992, 1.011)	
Divorced	0.986 (0.978, 0.994)		0.985 (0.977, 0.993)		0.986 (0.977, 0.994)	
Separated	0.994 (0.979, 1.009)		0.993 (0.978, 1.009)		0.994 (0.978, 1.010)	
Never married	0.994 (0.985, 1.003)		0.990 (0.981, 0.999)		0.996 (0.986, 1.006)	
Living with partner	0.997 (0.986, 1.009)		0.990 (0.978, 1.002)		0.996 (0.984, 1.008)	
Poverty Income Ratio		0.403		0.426		0.825
≤ 1.3	0.994 (0.989, 1.000)		0.992 (0.987, 0.997)		0.992 (0.987, 0.998)	
>1.3 and ≤ 3.5	0.992 (0.988, 0.997)		0.988 (0.983, 0.993)		0.990 (0.985, 0.995)	
>3.5	0.989 (0.984, 0.994)		0.986 (0.980, 0.991)		0.989 (0.983, 0.995)	
Missing	0.996 (0.987, 1.006)		0.989 (0.980, 0.999)		0.992 (0.982, 1.002)	
BMI (kg/m^2^)		0.980		0.912		0.938
<25	0.993 (0.987, 0.999)		0.988 (0.982, 0.994)		0.990 (0.983, 0.997)	
25–30	0.988 (0.960, 1.018)		0.984 (0.955, 1.015)		0.980 (0.947, 1.014)	
≥30	0.994 (0.989, 0.999)		0.990 (0.985, 0.995)		0.991 (0.986, 0.996)	
Missing	0.994 (0.990, 0.998)		0.990 (0.985, 0.994)		0.991 (0.986, 0.995)	
Smoking		0.787		0.665		0.382
Non-smoker	0.994 (0.990, 0.998)		0.989 (0.985, 0.993)		0.992 (0.988, 0.996)	
Smoker	0.993 (0.989, 0.997)		0.988 (0.984, 0.992)		0.989 (0.985, 0.993)	
Alcohol		0.612		0.354		0.432
Non-drinker	0.995 (0.989, 1.000)		0.992 (0.986, 0.998)		0.994 (0.988, 1.000)	
Drinker	0.992 (0.988, 0.996)		0.987 (0.983, 0.991)		0.989 (0.985, 0.993)	
Missing	0.991 (0.984, 0.997)		0.988 (0.981, 0.994)		0.990 (0.984, 0.997)	
Moderate activity		0.893		0.709		0.789
No	0.993 (0.990, 0.997)		0.989 (0.986, 0.993)		0.990 (0.986, 0.994)	
Yes	0.994 (0.989, 0.998)		0.988 (0.984, 0.993)		0.991 (0.986, 0.996)	
Vigorous activity		0.700		0.039		0.039
No	0.994 (0.991, 0.997)		0.990 (0.987, 0.993)		0.992 (0.989, 0.995)	
Yes	0.992 (0.985, 0.999)		0.982 (0.975, 0.989)		0.984 (0.976, 0.991)	
Gout		0.647		0.262		0.556
No	0.992 (0.989, 0.995)		0.988 (0.985, 0.991)		0.990 (0.987, 0.993)	
Yes	0.995 (0.985, 1.005)		0.994 (0.984, 1.004)		0.994 (0.983, 1.004)	
Congestive heart failure		0.770		0.396		0.437
No	0.992 (0.989, 0.995)		0.988 (0.985, 0.991)		0.990 (0.987, 0.993)	
Yes	0.994 (0.982, 1.006)		0.993 (0.981, 1.006)		0.995 (0.983, 1.008)	
Cancer		0.638		0.270		0.433
No	0.991 (0.988, 0.995)		0.987 (0.984, 0.991)		0.990 (0.987, 0.993)	
Yes	0.993 (0.986, 1.000)		0.992 (0.985, 0.999)		0.993 (0.986, 1.001)	
High blood pressure		0.654		0.404		0.527
No	0.992 (0.988, 0.996)		0.988 (0.984, 0.992)		0.990 (0.986, 0.994)	
Yes	0.991 (0.987, 0.995)		0.990 (0.986, 0.994)		0.992 (0.987, 0.996)	
Diabetes		0.516		0.183		0.120
No	0.992 (0.989, 0.995)		0.988 (0.984, 0.991)		0.989 (0.986, 0.993)	
Yes	0.992 (0.986, 0.998)		0.993 (0.987, 0.999)		0.996 (0.989, 1.002)	
Missing	0.983 (0.967, 0.999)		0.981 (0.964, 0.997)		0.981 (0.964, 0.998)	

a *Adjusted for: None*.

b *Adjusted for: gender, age, race*.

c *Adjusted for: gender, age, race, poverty income ratio, BMI, education, marital status, smoking, alcohol, energy, vigorous activity, moderate activity, gout, diabetes, high blood pressure, congestive heart failure, cancer. All the models are not adjusted for the variable itself in each stratification*.

### Sensitivity Analyses

This study used multivariate logistic regression to perform sensitivity analyses after removing extreme HEI-2015 scores and extreme energy intake values and calculating HEI-2015 based on day 2 dietary recall data. All results showed that HEI-2015 was negatively correlated with urolithiasis in the non-adjusted model, the minimally adjusted model, and the fully adjusted model, which was consistent with the above findings. The results of sensitivity analyses were presented in Supplementary Tables S4–S6.

## Discussion

This cross-sectional study explored the association between HEI-2015 and the prevalence of kidney stones by analyzing 6 cycles of NHANES data sets. Results showed that a higher HEI-2015 score was significantly associated with a lower prevalence of nephrolithiasis. Namely, a better diet quality was significantly correlated to a lower risk of kidney calculi. Education level and vigorous activity can modify the association. Of the 13 HEI-2015 components, scores of added sugars, total fruits, total vegetables and whole fruits were the most associated with kidney stones.

Diet is closely related to kidney diseases (9, 10). At the same time, diet plays an important role in the development of kidney stones, especially the intake of calcium, sodium, fructose, water and other beverages (11).

Our results show that better compliance with HEI-2015, namely better diet quality, is negatively related to the risk of kidney stones. However, there are few studies to clarify the relationship between diet quality (adherence to HEI-2015) and kidney stones. Many of the existing health problems (e.g., overweight, obesity, metabolic syndrome, and diabetes) are closely related to diet (12). In addition, diet is related to metabolic syndrome, as an active and healthy diet is the first-line intervention (13), and the Mediterranean diet can reduce the occurrence of metabolic syndrome (14, 15). Reportedly, improved diet quality reduces the risk of type 2 diabetes, and is associated with a lower risk of diabetes (16). Metabolic diseases such as obesity, metabolic syndrome, and diabetes are risk factors for the formation of kidney stones (17–19).

For HEI-2015, better diet quality means higher consumption of adequacy components (total fruits, whole fruits, total vegetables, greens and beans, whole grains, dairy, total protein foods, seafood and plant proteins, fatty acids) and lower consumption of moderation components (sodium, refined grains, added sugars, saturated fats). Our results indicate that sufficient total fruits, whole fruits, total vegetables and less added sugars are all associated with a lower risk of kidney stones. Reportedly, a diet rich in fruits and vegetables is associated with reduced kidney injury and a lower risk of CKD progression (20), and kidney injury is a factor closely related to the formation of nephrolithiasis. Currently, there are still few articles on the direct relationship between added sugar and kidney stones, but studies show that excessive consumption of added sugar is a risk factor for cardiometabolic diseases (including obesity, type 2 diabetes, and cardiovascular disease) (21), and these factors are closely related to the formation of kidney calculi. Therefore, these indirect evidences also show that better diet quality is associated with a lower risk of kidney stones, which is consistent with our results. Nevertheless, the specific mechanisms and the causal relationship between them shall be further clarified by more high-quality basic and prospective research.

We find that education level and vigorous activity can modify the association between HEI-2015 and kidney stones. Among the participants with higher education level and vigorous activity, the negative relationship between HEI-2015 and the risk of kidney stones is more obvious. We guess that those with higher education level may more closely adhere to a higher HEI-2015 and be more likely to benefit, which still requires further research. As reported, education and lifestyle play important roles in preventing the formation of stones (22). Also, the combination of regular physical activity and a healthy diet is associated with better health outcomes compared to the single effects (23), which is consistent with our results. In other words, among people with high education levels and vigorous activity, better diet quality is associated with lower risk of kidney stones. The above discussion is only speculations based on the existing literature and our research results, and calls for more high-quality research to further elucidate the underlying relationship. Nevertheless, what we can determine at present is that the overall trend is consistent regardless of the effect size (effect values are all < 1), or namely HEI-2015 is inversely associated with the prevalence of nephrolithiasis in any education level and any vigorous activity status.

This study is based on a large population analysis about the association between diet quality (based on HEI-2015) and the prevalence of kidney stones, although it is a cross-sectional study rather than a prospective one. In addition, compared with a certain nutrient element, HEI-2015 is a more comprehensive nutritional assessment method, which can better represent the comprehensive nutritional intake of an individual. Therefore, analyzing the relationship between HEI-2015 and kidney stones can more comprehensively show the relationship between nutrition or dietary intake and kidney stones. Furthermore, this research provides a convenient and practical insight into the prevention and control of nephrolithiasis, namely, reducing the prevalence of nephrolithiasis by adhering to better diet quality. Based on a multivariate regression analysis on the components of HEI-2015, we can further understand the relationship between diet quality and kidney stones.

But there are still some limitations. First, we cannot draw a causal relationship between diet quality and the risk of kidney stones due to the cross-sectional study design. Additionally, despite the adjustment of some potential confounding factors, we still cannot completely exclude the confounding caused by some unknown variables. Furthermore, the data on kidney stones came from questionnaires. Some participants were unwilling to answer related questions for a variety of reasons, resulting in non-response, and some participants may have missed some information when answering the questionnaire, all of which will result in bias. In addition, there is no relevant data on kidney stone nature, size, and treatment history in the NHANES database, so we cannot obtain the relationship between different types of kidney stones and HEI-2015 scores, which is also a limitation of this study.

## Conclusion

HEI-2015 is inversely associated with the prevalence of kidney stones, which means that better diet quality is associated with a lower risk of nephrolithiasis. This negative relationship is more obvious among people with high-level education and those with vigorous activity. Nevertheless, more high-quality prospective studies are needed to clarify the causal relationship between them.

## Data Availability Statement

The original contributions presented in the study are included in the article/Supplementary Material, further inquiries can be directed to the corresponding author.

## Ethics Statement

The studies involving human participants were reviewed and approved by National Center for Health Statistics (NCHS) research ethics review board. The patients/participants provided their written informed consent to participate in this study.

## Author Contributions

JiaW and SY: conceptualization and methodology. SY, JiahW, and ZY: data acquisition. SY, JiahW, YB, ZY, JC, and YX: software and formal analysis. SY and JiahW: writing—original draft. JiaW: data curation and supervision. All authors contributed in writing—review and editing, read, and approved the final manuscript.

## Funding

This work was supported by the 1.3.5 project for disciplines of excellence, West China Hospital, Sichuan University (Grant No. ZY2016104).

## Conflict of Interest

The authors declare that the research was conducted in the absence of any commercial or financial relationships that could be construed as a potential conflict of interest.

## Publisher's Note

All claims expressed in this article are solely those of the authors and do not necessarily represent those of their affiliated organizations, or those of the publisher, the editors and the reviewers. Any product that may be evaluated in this article, or claim that may be made by its manufacturer, is not guaranteed or endorsed by the publisher.

## Supplementary Material

Supplementary Figure S1 **(A–F)** The ratios of mean scores of HEI-2015 components to maximum scores (**A:** 2007–2008 cycle, **B:** 2009–2010 cycle, **C:** 2011–2012 cycle, **D:** 2013–2014 cycle, **E:** 2015–2016 cycle, **F:** 2017–2018 cycle), **(G)** Prevalence of kidney stones and HEI-2015 mean scores in each NHANES cycle.

Supplementary Table S1 HEI−2015 components and scoring standards.

Supplementary Table S2 Characteristics of participants by categories of kidney stones: NHANES 2007–2018.

Supplementary Table S3 Distribution of HEI-2015 components by categories of HEI-2015: NHANES 2007–2018.

Supplementary Table S4 Association of Healthy Eating Index 2015 with kidney stones (after removing extreme HEI-2015 scores).

Supplementary Table S5 Association of Healthy Eating Index 2015 with kidney stones (after removing extreme energy intake value).

Supplementary Table S6 Association of Healthy Eating Index 2015 (day 2 dietary recall) with kidney stones.

## Abbreviations

HEI: Healthy Eating Index
NHANES: National Health and Nutrition Examination Survey
BMI: body mass index.
