# Supplementary material for: Association Between Healthy Eating Index-2015 and Kidney Stones in American Adults: A Cross-Sectional Analysis of NHANES 2007–2018
Source: Front Nutr. 2022 May 24;9:820190. doi: 10.3389/fnut.2022.820190 (PMC9172846; doi:10.3389/fnut.2022.820190)
Supplement: Supplementary Figure S1 — (A–F) The ratios of mean scores of HEI-2015 components to maximum scores (A: 2007–2008 cycle, B: 2009–2010 cycle, C: 2011–2012 cycle, D: 2013–2014 cycle, E: 2015–2016 cycle, F: 2017–2018 cycle), (G) Prevalence of kidney stones and HEI-2015 mean scores in each NHANES cycle. [file Data_Sheet_1.zip › Table S3.docx]

**Table S3** Distribution of HEI-2015 Components by categories of HEI-2015: NHANES 2007–2018

| HEI-2015 Components | Q1(8.81-40.89) | Q2(40.89-50.33) | Q3(50.34-60.35) | Q4(60.35-97.88) | P value |
| --- | --- | --- | --- | --- | --- |
| Total Vegetables | 2.23 ± 1.56 | 2.88 ± 1.64 | 3.29 ± 1.61 | 3.76 ± 1.49 | <0.001 |
| Greens and Beans | 0.47 ± 1.30 | 1.13 ± 1.92 | 1.73 ± 2.22 | 2.77 ± 2.32 | <0.001 |
| Total Fruits | 0.65 ± 1.30 | 1.48 ± 1.85 | 2.41 ± 2.04 | 3.60 ± 1.78 | <0.001 |
| Whole Fruits | 0.57 ± 1.34 | 1.47 ± 2.03 | 2.42 ± 2.25 | 3.80 ± 1.90 | <0.001 |
| Whole Grains | 0.74 ± 1.65 | 1.62 ± 2.53 | 2.74 ± 3.30 | 4.89 ± 3.80 | <0.001 |
| Dairy | 5.07 ± 3.38 | 4.87 ± 3.42 | 4.94 ± 3.39 | 5.34 ± 3.39 | <0.001 |
| Total Protein Foods | 3.85 ± 1.47 | 4.18 ± 1.28 | 4.30 ± 1.22 | 4.52 ± 1.02 | <0.001 |
| Seafood and Plant Proteins | 0.89 ± 1.61 | 1.90 ± 2.17 | 2.70 ± 2.26 | 3.84 ± 1.90 | <0.001 |
| Fatty Acids | 2.78 ± 2.86 | 4.50 ± 3.47 | 5.59 ± 3.53 | 7.19 ± 3.27 | <0.001 |
| Sodium | 3.52 ± 3.32 | 4.18 ± 3.48 | 4.51 ± 3.51 | 5.32 ± 3.56 | <0.001 |
| Refined Grains | 4.14 ± 3.62 | 5.80 ± 3.67 | 6.82 ± 3.42 | 8.12 ± 2.68 | <0.001 |
| Saturated Fats | 3.88 ± 3.37 | 5.38 ± 3.49 | 6.47 ± 3.25 | 7.83 ± 2.64 | <0.001 |
| Added Sugars | 5.11 ± 3.69 | 6.30 ± 3.52 | 7.13 ± 3.16 | 8.26 ± 2.35 | <0.001 |
