# Supplementary material for: Association Between Healthy Eating Index-2015 and Kidney Stones in American Adults: A Cross-Sectional Analysis of NHANES 2007–2018
Source: Front Nutr. 2022 May 24;9:820190. doi: 10.3389/fnut.2022.820190 (PMC9172846; doi:10.3389/fnut.2022.820190)
Supplement: Supplementary Figure S1 — (A–F) The ratios of mean scores of HEI-2015 components to maximum scores (A: 2007–2008 cycle, B: 2009–2010 cycle, C: 2011–2012 cycle, D: 2013–2014 cycle, E: 2015–2016 cycle, F: 2017–2018 cycle), (G) Prevalence of kidney stones and HEI-2015 mean scores in each NHANES cycle. [file Data_Sheet_1.zip › Table S4.docx]

**Table S4** Association of Healthy Eating Index 2015 with kidney stones (after removing extreme HEI-2015 scores)

| Exposure | Model 1^a^ | Model 2^b^ | Model 3^c^ |
| --- | --- | --- | --- |
| HEI-2015 (continuous) | 0.991 (0.987, 0.994) <0.001 | 0.986 (0.983, 0.990) <0.001 | 0.989 (0.986, 0.993) <0.001 |
| Quartile of HEI-2015 |  |  |  |
| Q1 (10.000-40.881) | 1.0 | 1.0 | 1.0 |
| Q2 (40.883-50.324) | 1.029 (0.883, 1.199) 0.716 | 0.961 (0.827, 1.117) 0.606 | 0.993 (0.854, 1.155) 0.926 |
| Q3 (50.325-60.316) | 0.905 (0.785, 1.043) 0.172 | 0.806 (0.697, 0.933) 0.005 | 0.854 (0.735, 0.992) 0.043 |
| Q4 (60.316-92.181) | 0.748 (0.643, 0.870) <0.001 | 0.632 (0.544, 0.735) <0.001 | 0.713 (0.608, 0.836) <0.001 |
| P value for trend | <0.001 | <0.001 | <0.001 |

^a^ Non-adjusted model: adjusted for None
^b^ Minimally adjusted model: adjusted for gender, age, race

^c^ Fully adjusted model: adjusted for gender, age, race, poverty income ratio, BMI, education, marital status, smoking, alcohol, energy, vigorous activity, moderate activity, gout, diabetes, high blood pressure, congestive heart failure, cancer
