# Supplementary material for: Association Between Healthy Eating Index-2015 and Kidney Stones in American Adults: A Cross-Sectional Analysis of NHANES 2007–2018
Source: Front Nutr. 2022 May 24;9:820190. doi: 10.3389/fnut.2022.820190 (PMC9172846; doi:10.3389/fnut.2022.820190)
Supplement: Supplementary Figure S1 — (A–F) The ratios of mean scores of HEI-2015 components to maximum scores (A: 2007–2008 cycle, B: 2009–2010 cycle, C: 2011–2012 cycle, D: 2013–2014 cycle, E: 2015–2016 cycle, F: 2017–2018 cycle), (G) Prevalence of kidney stones and HEI-2015 mean scores in each NHANES cycle. [file Data_Sheet_1.zip › Table S5.docx]

**Table S5** Association of Healthy Eating Index 2015 with kidney stones (after removing extreme energy intake value)

| Exposure | Model 1^a^ | Model 2^b^ | Model 3^c^ |
| --- | --- | --- | --- |
| HEI-2015 (continuous) | 0.990 (0.986, 0.994) <0.001 | 0.985 (0.981, 0.989) <0.001 | 0.989 (0.985, 0.992) <0.001 |
| Quartile of HEI-2015 |  |  |  |
| Q1 (8.808-41.144) | 1.0 | 1.0 | 1.0 |
| Q2 (41.144-50.642) | 1.025 (0.877, 1.197) 0.759 | 0.951 (0.817, 1.108) 0.523 | 0.994 (0.852, 1.160) 0.941 |
| Q3 (50.643-60.703) | 0.909 (0.785, 1.054) 0.209 | 0.806 (0.691, 0.941) 0.008 | 0.857 (0.733, 1.003) 0.059 |
| Q4 (60.703-97.877) | 0.721 (0.617, 0.842) <0.001 | 0.606 (0.520, 0.708) <0.001 | 0.694 (0.593, 0.812) <0.001 |
| P value for trend | <0.001 | <0.001 | <0.001 |

^a^ Non-adjusted model: adjusted for None
^b^ Minimally adjusted model: adjusted for gender, age, race

^c^ Fully adjusted model: adjusted for gender, age, race, poverty income ratio, BMI, education, marital status, smoking, alcohol, energy, vigorous activity, moderate activity, gout, diabetes, high blood pressure, congestive heart failure, cancer
