# Supplementary material for: Association Between Healthy Eating Index-2015 and Kidney Stones in American Adults: A Cross-Sectional Analysis of NHANES 2007–2018
Source: Front Nutr. 2022 May 24;9:820190. doi: 10.3389/fnut.2022.820190 (PMC9172846; doi:10.3389/fnut.2022.820190)
Supplement: Supplementary Figure S1 — (A–F) The ratios of mean scores of HEI-2015 components to maximum scores (A: 2007–2008 cycle, B: 2009–2010 cycle, C: 2011–2012 cycle, D: 2013–2014 cycle, E: 2015–2016 cycle, F: 2017–2018 cycle), (G) Prevalence of kidney stones and HEI-2015 mean scores in each NHANES cycle. [file Data_Sheet_1.zip › Table S6.docx]

**Table S6** Association of Healthy Eating Index 2015 (day 2 dietary recall) with kidney stones

| Exposure | Model 1^a^ | Model 2^b^ | Model 3^c^ |
| --- | --- | --- | --- |
| HEI-2015 (continuous) | 0.994 (0.991, 0.998) 0.002 | 0.990 (0.986, 0.994) <0.001 | 0.993 (0.989, 0.998) 0.004 |
| Quartile of HEI-2015 |  |  |  |
| Q1 (0.000-41.614) | 1.0 | 1.0 | 1.0 |
| Q2 (41.616-51.381) | 0.907 (0.768, 1.071) 0.251 | 0.826 (0.699,0.976) 0.028 | 0.869 (0.733, 1.030) 0.112 |
| Q3 (51.383-62.135) | 0.902 (0.779, 1.044) 0.168 | 0.799 (0.688, 0.928) 0.004 | 0.867 (0.737, 1.020) 0.092 |
| Q4 (62.136-98.502) | 0.790 (0.683, 0.914) 0.002 | 0.668 (0.572, 0.780) <0.001 | 0.759 (0.640, 0.901) 0.003 |
| P value for trend | 0.002 | <0.001 | 0.004 |

^a^ Non-adjusted model: adjusted for None
^b^ Minimally adjusted model: adjusted for gender, age, race

^c^ Fully adjusted model: adjusted for gender, age, race, poverty income ratio, BMI, education, marital status, smoking, alcohol, energy, vigorous activity, moderate activity, gout, diabetes, high blood pressure, congestive heart failure, cancer
