# Supplementary material for: Association Between Healthy Eating Index-2015 and Kidney Stones in American Adults: A Cross-Sectional Analysis of NHANES 2007–2018
Source: Front Nutr. 2022 May 24;9:820190. doi: 10.3389/fnut.2022.820190 (PMC9172846; doi:10.3389/fnut.2022.820190)
Supplement: Supplementary Figure S1 — (A–F) The ratios of mean scores of HEI-2015 components to maximum scores (A: 2007–2008 cycle, B: 2009–2010 cycle, C: 2011–2012 cycle, D: 2013–2014 cycle, E: 2015–2016 cycle, F: 2017–2018 cycle), (G) Prevalence of kidney stones and HEI-2015 mean scores in each NHANES cycle. [file Data_Sheet_1.zip › Table S2.docx]

**Table S2** Characteristics of participants by categories of kidney stones: NHANES 2007–2018

|  | Without kidney stones | With kidney stones | P value |
| --- | --- | --- | --- |
| Energy (kcal) | 2173.04 ± 995.39 | 2131.31 ± 947.36 | 0.028 |
| HEI_2015 | 51.00 ± 13.88 | 49.24 ± 13.01 | <0.001 |
| Age (years, mean ± SD) | 47.03 ± 16.87 | 53.60 ± 15.45 | <0.001 |
| 20-34 (%) | 28.40 | 13.18 |  |
| 35-49 (%) | 27.60 | 25.93 |  |
| 50-64 (%) | 26.29 | 34.90 |  |
| ≥65 (%) | 17.70 | 26.00 |  |
| Gender (%) |  |  | <0.001 |
| Male | 47.99 | 54.57 |  |
| Female | 52.01 | 45.43 |  |
| Race (%) |  |  | <0.001 |
| Mexican American | 8.59 | 5.98 |  |
| Other Hispanic | 5.77 | 5.04 |  |
| Non-Hispanic white | 66.10 | 77.17 |  |
| Non-Hispanic black | 11.77 | 5.91 |  |
| Other races | 7.77 | 5.90 |  |
| Education (%) |  |  | 0.025 |
| Less than 9th grade | 5.17 | 4.78 |  |
| 9-11th grade | 10.28 | 10.71 |  |
| High school graduate | 23.15 | 23.10 |  |
| Some college | 31.22 | 33.57 |  |
| College graduate or above | 30.18 | 27.84 |  |
| Marital Status (%) |  |  | <0.001 |
| Married | 54.29 | 63.03 |  |
| Widowed | 5.52 | 6.41 |  |
| Divorced | 10.08 | 12.31 |  |
| Separated | 2.42 | 2.23 |  |
| Never married | 19.31 | 9.93 |  |
| Living with partner | 8.38 | 6.08 |  |
| Poverty Income Ratio (mean ± SD) | 3.00 ± 1.65 | 3.05 ± 1.61 | 0.131 |
| Poverty Income Ratio (category, %) |  |  | 0.008 |
| ≤1.3 | 20.23 | 18.04 |  |
| >1.3 and ≤3.5 | 32.85 | 35.18 |  |
| >3.5 | 39.65 | 39.93 |  |
| Missing | 7.27 | 6.85 |  |
| BMI (kg/m^2^, mean ± SD) | 28.96 ± 6.89 | 30.65 ± 6.96 | <0.001 |
| <25 (%) | 28.87 | 18.59 |  |
| 25-30 (%) | 1.63 | 0.87 |  |
| ≥30 (%) | 32.70 | 32.57 |  |
| Missing (%) | 36.80 | 47.98 |  |
| Smoking (%) |  |  | <0.001 |
| Non-smoker | 56.25 | 50.61 |  |
| Smoker | 43.75 | 49.39 |  |
| Alcohol (%) |  |  | 0.464 |
| Non-drinker | 17.67 | 18.57 |  |
| Drinker | 60.72 | 59.95 |  |
| Missing | 21.60 | 21.48 |  |
| High Blood Pressure (%) |  |  | <0.001 |
| No | 69.32 | 52.81 |  |
| Yes | 30.68 | 47.19 |  |
| Diabetes (%) |  |  | <0.001 |
| No | 89.02 | 77.89 |  |
| Yes | 8.94 | 18.43 |  |
| Missing | 2.04 | 3.68 |  |
| Congestive Heart Failure (%) |  |  | <0.001 |
| No | 97.87 | 95.46 |  |
| Yes | 2.13 | 4.54 |  |
| Cancer (%) |  |  | <0.001 |
| No | 90.18 | 83.85 |  |
| Yes | 9.82 | 16.15 |  |
| Gout (%) |  |  | <0.001 |
| No | 96.33 | 91.86 |  |
| Yes | 3.67 | 8.14 |  |
| Vigorous activity (%) |  |  | <0.001 |
| No | 73.18 | 81.25 |  |
| Yes | 26.82 | 18.75 |  |
| Moderate activity (%) |  |  | <0.001 |
| No | 53.69 | 58.70 |  |
| Yes | 46.31 | 41.30 |  |

Mean ± SD for continuous variables, P value from weighted t test.

% for categorical variables, P value from weighted Chi-square test.
