# Supplementary figures and images for: Association Between Healthy Eating Index-2015 and Kidney Stones in American Adults: A Cross-Sectional Analysis of NHANES 2007–2018
Source: Front Nutr. 2022 May 24;9:820190. doi: 10.3389/fnut.2022.820190 (PMC9172846; doi:10.3389/fnut.2022.820190)

A

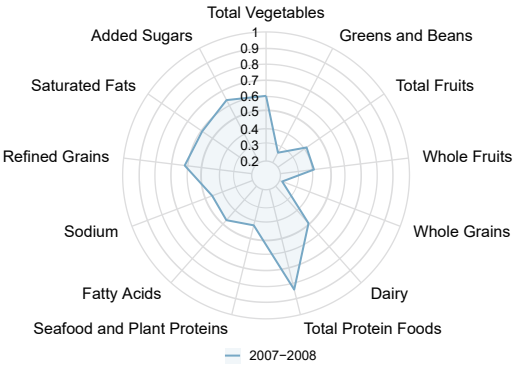

B

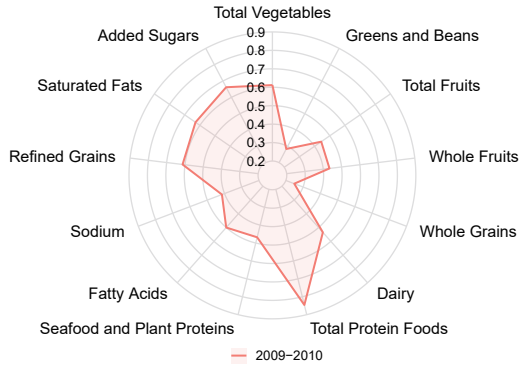

C

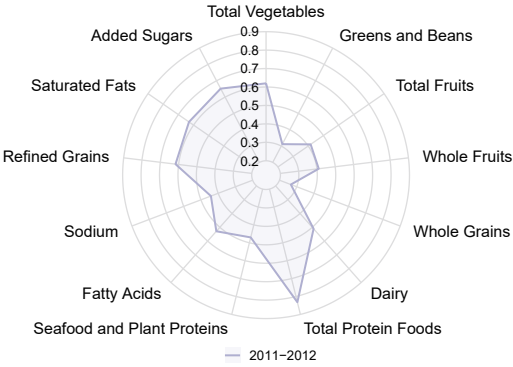

D

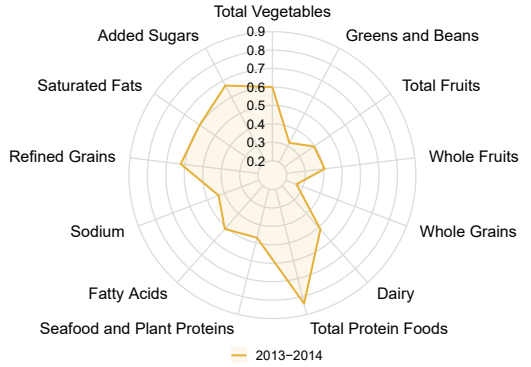

E

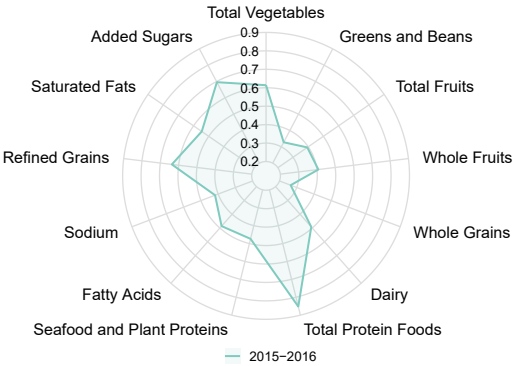

F

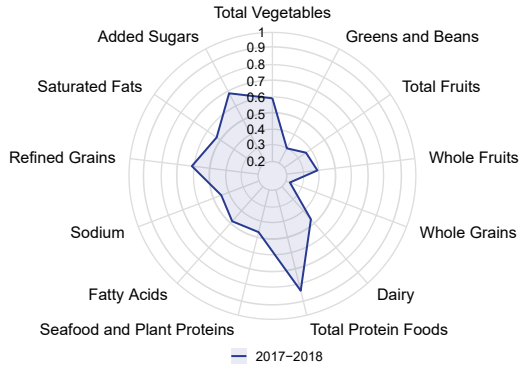

G

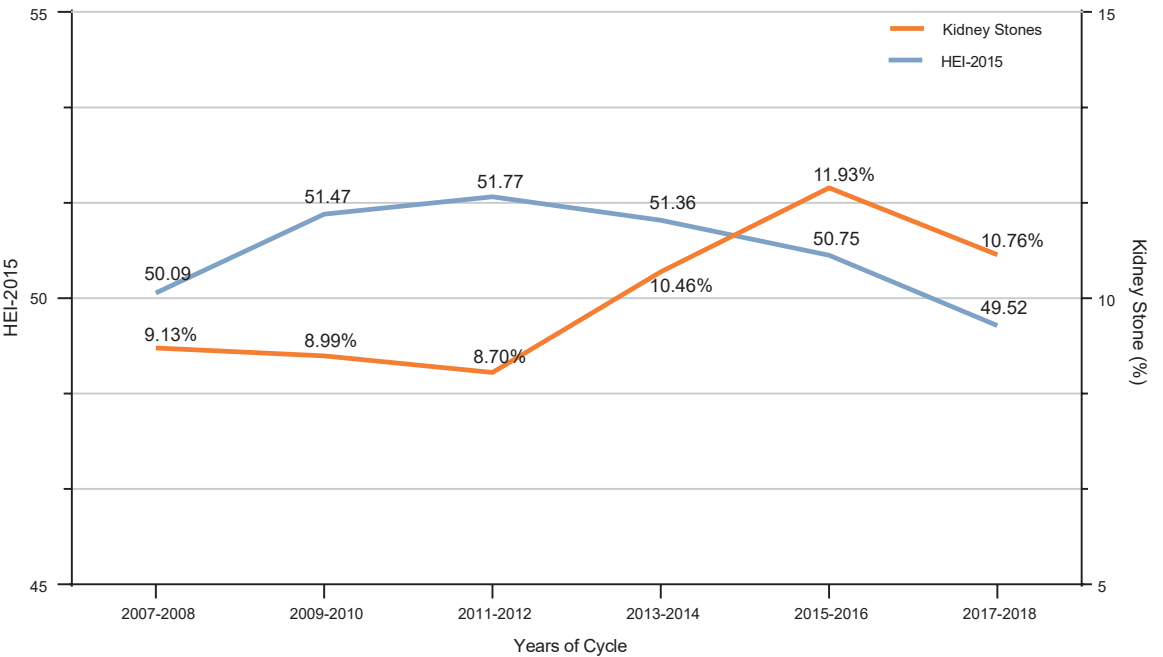

Supplement: Supplementary Figure S1 — (A–F) The ratios of mean scores of HEI-2015 components to maximum scores (A: 2007–2008 cycle, B: 2009–2010 cycle, C: 2011–2012 cycle, D: 2013–2014 cycle, E: 2015–2016 cycle, F: 2017–2018 cycle), (G) Prevalence of kidney stones and HEI-2015 mean scores in each NHANES cycle. [file Data_Sheet_1.zip › Figure S1.pdf]
